# Long-term oral glucocorticoid use is associated with complications, healthcare resource utilization, and costs among patients with dermatomyositis or polymyositis

**DOI:** 10.1007/s10067-025-07651-1

**Published:** 2025-09-10

**Authors:** Rohit Aggarwal, Qian Cai, Daniel Labson, Concetta Crivera, Federico Zazzetti

**Affiliations:** 1 Division of Rheumatology and Clinical Immunology, University of Pittsburgh School of Medicine, Pittsburgh, PA USA; 2 Global Market Access RWE, Johnson & Johnson, Titusville, NJ USA; 3 Global Market Access RWE, Johnson & Johnson, Raritan, NJ USA; 4 Immunology Market Access, Johnson & Johnson, Horsham, PA USA; 5 Immunology Medical Affairs, Johnson & Johnson, Horsham, PA USA

**Keywords:** Dermatomyositis, Healthcare resource utilization, Long-term steroid use, Oral glucocorticoids, Polymyositis, Real-world data

## Abstract

**Introduction/Objective:**

Oral glucocorticoids (OGC) are conventionally used as first-line treatment for dermatomyositis (DM) and polymyositis (PM). This study evaluated clinical and economic outcomes associated with long-term (LT) OGC use in DM/PM.

**Methods:**

Adults with ≥ 2 medical claims of DM/PM 30‒365 days apart from January 1, 2016, to December 31, 2022, and ≥ 1 diagnosis code of a physician specialty of interest were selected from the MarketScan Commercial and Medicare Supplemental databases. Patients should have ≥ 1 OGC pharmacy claim (i.e., date of first claim defined as index date) on or after the diagnosis date, ≥ 12 months pre- and post-index continuous enrollment, and no diagnosis of inclusion body myositis during the study. The LT group included patients with continuous OGC use for ≥ 3 consecutive months within the 12-month post-index period, whereas short-term (ST) users included those with < 3 consecutive months of OGC use.

**Results:**

Two thousand two-hundred eighty patients were included (LT, 1313 [57.6%]; ST, 967 [42.4%]). Compared with ST, LT OGC patients had significantly higher incidences of OGC-related complications, such as heart failure (adjusted odds ratio [aOR], 1.8; 95% CI, 1.3‒2.7) and osteoporosis (aOR, 1.9; 95% CI, 1.4‒2.6), after covariate adjustment. LT users had increased odds for both all-cause (aOR, 1.7; 95% CI, 1.3‒2.2) and disease-related inpatient admission (aOR, 3.8; 95% CI, 2.3‒6.2) versus ST users. Higher adjusted average annual all-cause (by US $30,555; *p* < 0.01) and disease-related costs (by US $21,311; *p* < 0.01) were incurred by LT versus ST users.

**Conclusions:**

DM/PM patients with LT OGC use had higher rates of associated medical conditions and higher healthcare resource utilization and costs than ST users.

**Table:** 

Key points • Long-term use of oral glucocorticoids (OGC) in patients with dermatomyositis (DM) and polymyositis (PM) is associated with increased risk of complications, and there is limited real-world evidence on the association of long-term use with clinical and economic outcomes. • This real-world claims-based study demonstrated that long-term OGC use in patients with DM/PM leads to higher rates of complications, advanced treatment use and healthcare resource utilization, and higher costs compared with short-term OGC use. • These findings highlight the limitations of OGC as a therapeutic tool and further support an unmet medical need for new treatment options among patients with DM/PM. • Disease management with novel treatments that have an advantageous safety profile and can be utilized any time throughout the disease course may provide safer, more efficacious alternatives to OGC.

## Introduction

Idiopathic inflammatory myopathy (IIM) is a heterogeneous group of rare autoimmune conditions mainly characterized by muscle weakness and inflammation [1–3]. Disease classification has evolved over the years for IIM, with the original classification system including two major types of disease among others: dermatomyositis (DM) and polymyositis (PM) [1–3]. More recently, IIM has been classified into four major types based on myositis-specific antibodies and a comprehensive clinicopathological approach: DM, immune-mediated necrotizing myopathy, anti-synthetase syndrome, and inclusion body myositis (IBM) [4], with the status of PM as a separate entity currently considered unclear [1, 3, 4]. However, there are limitations to the identification of some IIM subtypes in retrospective studies, such as anti-synthetase syndrome and immune-mediated necrotizing myopathy, as these subtypes may not have specific diagnosis codes.

Due to its low incidence, IIM is considered a rare disease, although epidemiology estimates for IIM can be complicated by differences in study design and estimation methods across studies. As such, the reported incidence of IIM ranges from 0.2 to 2 per 100,000 person-years (PY) and prevalence from 2 to 25 per 100,000 PY [5]. Published data for PM and DM as distinct conditions indicate a higher incidence of PM (ranging from 2.5–3.8 per 100,000 PY in the USA) compared with DM (1.4–1.7 per 100,000 PY in the USA) [6–8].

One of the main treatment goals in patients with IIM is the achievement and maintenance of remission [9]. Oral glucocorticoids (OGC) are conventionally used as the first-line treatment for patients with IIM [2, 9, 10], but they are not always effective in inducing and maintaining remission and are associated with a wide range of medical conditions that increase with long-term (LT) use, including diabetes, hypertension, cardiovascular disease, bone fractures, psychiatric events, infections, and ophthalmic and gastrointestinal conditions [9–14]. Because OGC use is not effective for all patients, some patients with IIM need to use second-line therapies, which often include immunosuppressants or intravenous immunoglobulin (IVIG) [9, 12].

Previous studies have also shown that patients with IIM have a substantial healthcare resource utilization (HCRU) burden. A study using administrative data from Québec in Canada found that healthcare costs for patients with DM and PM were equivalent to or higher than other serious chronic autoimmune diseases (e.g., rheumatoid arthritis and systemic sclerosis) [15]. Another study using US claims-based data found that patients with IIM (defined as DM, PM, or interstitial myositis) had significantly higher medical costs compared with a matched control population (*p* < 0.001), with significant differences also observed separately for DM and PM [16]. These results were confirmed in a later US study that was focused specifically on patients with DM or PM, which again showed significantly higher HCRU for patients versus controls, as well as significantly more medically related work loss (*p* < 0.001 for both) [17]. In addition, LT OGC use independent of any disease is associated with substantial HCRU and costs [13, 18], which may further exacerbate the financial burden in patients with IIM who are receiving LT OGC treatment.

Although the overall HCRU burden of IIM has been reported in some studies, there is a dearth of literature specifically focused on clinical and economic outcomes associated with LT OGC use in this population. The goal of this retrospective, observational cohort study aimed at evaluating the impact of LT OGC use on incident OGC-related medical conditions as well as the association between LT OGC use and annual HCRU and healthcare costs among patients with DM or PM.

## Materials and methods

### Data sources

This retrospective, observational cohort study used administrative medical and pharmacy claims data derived from the MarketScan Commercial Claims and Encounters and Medicare Supplemental databases between January 1, 2016, and December 31, 2022. The databases contain fully paid and adjudicated inpatient, outpatient, and pharmacy insurance claims data of active employees, their spouses, and dependents covered by employer-sponsored private health insurance, and Medicare-eligible retirees covered by Medicare Supplemental health insurance plans in the USA [19]. Both databases provide detailed information on HCRU and associated costs for healthcare services performed in both inpatient and outpatient settings, as well as pharmacy services. Data records were de-identified and certified to be fully compliant with the Health Insurance Portability and Accountability Act patient confidentiality requirements.

### Study patients

Eligible patients were aged ≥ 18 years and had ≥ 2 medical claims of DM or PM (International Classification of Diseases, version 10, Clinical Modification [ICD-10-CM] starting with M33.0 or M33.1 [DM], or M33.2 or M33.9 [PM]), ≥ 30 but ≤ 365 days apart between January 1, 2016, and December 31, 2022, and ≥ 1 diagnosis code associated with a physician specialty of interest (i.e., rheumatologist, dermatologist, neurologist, pulmonologist, internal medicine, or general practitioner). Included patients were required to have ≥ 1 pharmacy claim for OGC on or after the diagnosis date. The date of the first OGC claim was designated as the index date. Patients included in the analyses had ≥ 12 months pre-index (baseline period) and ≥ 12 months post-index (follow-up period) continuous enrollment; patients with a diagnosis of IBM during the study period were excluded due to IBM being significantly different than other forms of IIM and not being treated with LT immunosuppressants [20]. Patients were classified into two groups: LT vs short-term (ST) OGC users. The LT OGC group included patients with OGC use for ≥ 3 consecutive months, defined as the number of days from the index date to discontinuation date (i.e., dispensing date of previous fill plus days’ supply of the last fill given a gap of 90 days) within the 12-month post-index period, regardless of dosage. Patients who did not satisfy these OGC-use duration criteria were classified into the ST group.

### Study measures

Associated OGC-related medical conditions of interest included cardiovascular diseases, ocular diseases, malignancies, psychiatric disorders, and other medical conditions (eg, Cushing syndrome). Subsequent treatments of interest included anti-inflammatory agents/antibiotics (dapsone, sulphapyridine, tetracyclines, doxycycline, minocycline), immunosuppressants (azathioprine, chlorambucil, cyclophosphamide, mycophenolate mofetil, cyclosporine, tacrolimus), rituximab, intravenous or subcutaneous immunoglobulin, anticomplement agents (eculizumab, ravulizumab), Janus kinase (JAK) inhibitors (tofacitinib, ruxolitinib, baricitinib), statins (atorvastatin, cerivastatin, fluvastatin, lovastatin, pitavastatin, pravastatin, rosuvastatin, simvastatin), other immunomodulators (omalizumab, dupilumab), and plasma exchange.

All-cause HCRU was defined as the use of resources associated with any condition incurred from inpatient and outpatient services, including emergency room visits, physician office visits, and other outpatient services, such as laboratory and radiology exams. Disease-related HCRU was defined as the use of resources associated with DM and PM diagnoses incurred from medical services. All-cause healthcare costs were defined as total payments incurred from fully adjudicated claims of prescriptions and medical services associated with any condition, whereas disease-related costs were defined as payments associated with DM and PM diagnoses or corresponding pharmacologic treatments. All costs were adjusted to 2022 values using the US Medical Care Component of the Consumer Price Index. All study measures were evaluated during the 12-month post-index period.

### Statistical analyses

Study measures were examined descriptively and compared between the LT and ST OGC groups. Means, standard deviation (SD), and medians were reported for continuous and count variables. Frequency and percentage were reported for categorical variables. To compare differences in patient characteristics and outcome measures between the two groups, chi-square tests or exact Fisher tests were used for categorical variables, and *t*-tests were used for continuous variables. Multivariable logistic regressions were performed to assess the association between duration of OGC and incident associated medical conditions or HCRU after adjusting for baseline characteristics. Odds ratios and 95% confidence intervals (CIs) are presented. Generalized linear models with log link function and gamma distribution were used to estimate the costs for between-group comparisons with adjustment for baseline characteristics. Differences were considered significant if *p* < 0.05. All data analyses were conducted using SAS 9.4 (SAS Institute, Cary, NC).

## Results

### Demographics and clinical characteristics

A total of 2280 patients were included, of whom 1313 (57.6%) were in the LT group and 967 (42.4%) were in the ST group (Table 1). Overall, the mean age was 53.2 (SD 13.4) years, 74.6% of patients were female, and 47.7% had a diagnosis code associated with a rheumatologist. LT OGC users had a higher Charlson comorbidity index than ST OGC users during the 12-month pre-index period (2.2 vs 1.9; *p* < 0.01). The most common pre-index conditions were hypertension (44.0% of the total population) and hyperlipidemia (34.5%).

**Table 1:** Baseline demographic and clinical characteristics of study patients

Characteristic	All patients (*N* = 2280)	LT OGC use (*n* = 1313)	ST OGC use (*n* = 967)	*p* value^a^
Age, years				< 0.01
Mean (SD)	53.2 (13.4)	54.6 (13.6)	51.3 (13.0)	
Gender, *n* (%)				0.02
Female	1702 (74.6)	957 (72.9)	745 (77.0)	
Region, *n* (%)				< 0.01
North Central	490 (21.5)	300 (22.8)	190 (19.6)	
Northeast	360 (15.8)	216 (16.5)	144 (14.9)	
South	1207 (52.9)	643 (49.0)	564 (58.3)	
West	223 (9.8)	154 (11.7)	69 (7.1)	
Most common diagnosing physician specialty, *n* (%)				< 0.01
Rheumatologist	1088 (47.7)	668 (50.9)	420 (43.4)	
Internal medicine	399 (17.5)	231 (17.6)	168 (17.4)	
General practitioner	349 (15.3)	164 (12.5)	185 (19.1)	
Follow-up, years, mean (SD)	4.6 (1.9)	4.4 (1.9)	4.9 (1.9)	< 0.01
12-month pre-index CCI, mean (SD)	2.1 (1.7)	2.2 (1.7)	1.9 (1.7)	< 0.01
Most common 12-month pre-index comorbidities, *n* (%)				< 0.01
Hypertension	1004 (44.0)	595 (45.3)	409 (42.3)	0.15
Hyperlipidemia	786 (34.5)	429 (32.7)	357 (36.9)	0.04
Arthralgia	753 (33.0)	428 (32.6)	325 (33.6)	0.61
Fatigue	625 (27.4)	374 (28.5)	251 (26.0)	0.18
Systemic lupus erythematosus	238 (10.4)	142 (10.8)	96 (9.9)	0.49
Rheumatoid arthritis	198 (8.7)	126 (9.6)	72 (7.4)	0.07
Sjögren’s syndrome	110 (4.8)	58 (4.4)	52 (5.4)	0.29
Treatment use, *n* (%)				
Glucocorticoids	1684 (73.9)	1136 (86.5)	548 (56.7)	< 0.01
Immunosuppressants	1036 (45.4)	675 (51.4)	361 (37.3)	< 0.01
Statins	405 (17.8)	243 (18.5)	162 (16.8)	0.28

*CCI* Charlson comorbidity index, *LT* long-term, *OGC* oral glucocorticoid, *SD* standard deviation, *ST* short-term

^a^Comparisons refer to LT versus ST OGC exposure

During the 12-month post-index period, the mean (SD)/median prednisone equivalent average daily dose was 18.8 (17.7)/13.6 mg, and the mean OGC persistence was 5.4 months.

### Incident-associated medical conditions

**Fig. 1:**
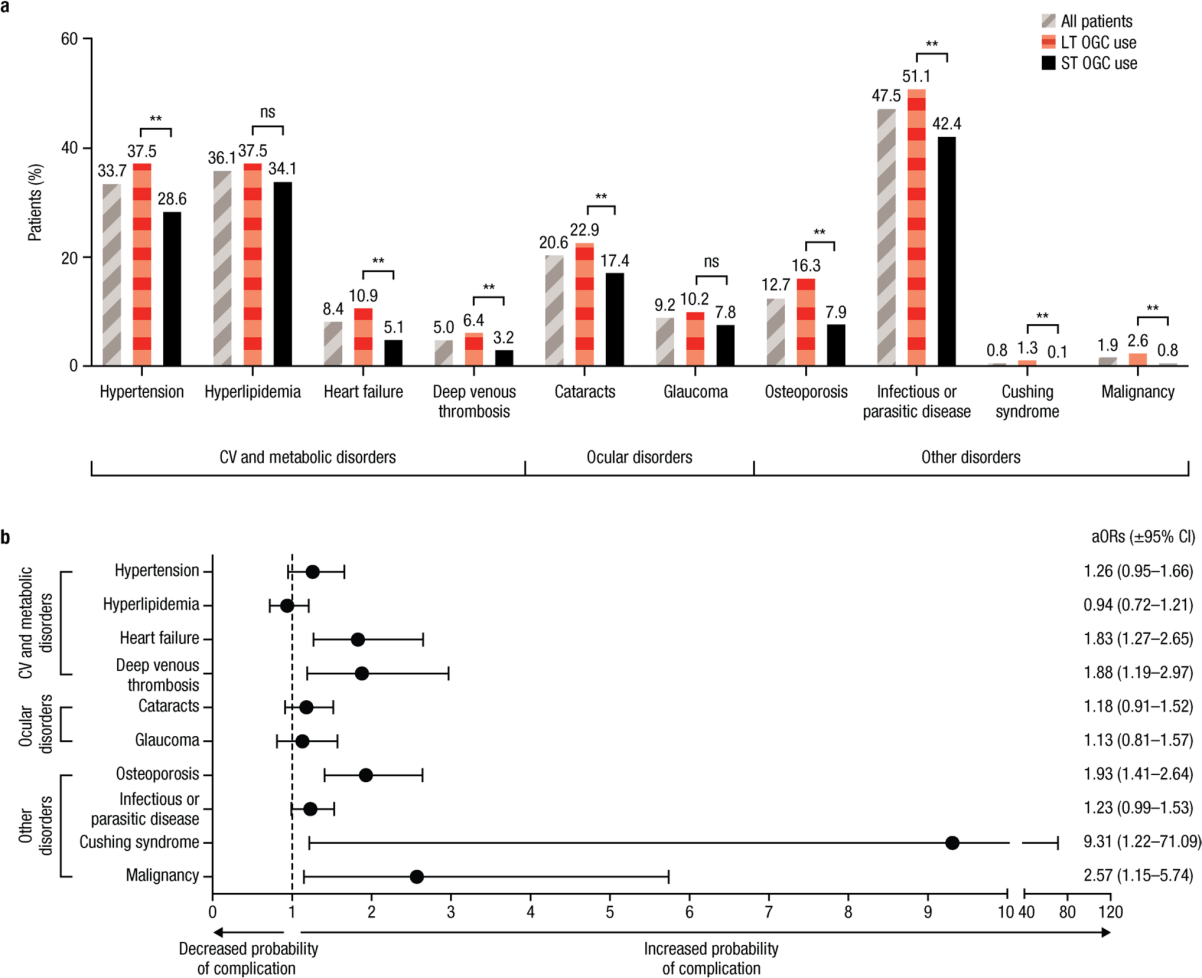
**a** Incidence of associated medical conditions of interest.** b** aORs (± 95% CI) in patients with LT versus ST OGC use during the 12-month post-index period. aOR, adjusted odd ratio; CI, confidence interval; CV, cardiovascular; LT, long-term; NS, not significant; OGC, oral glucocorticoid; ST, short-term. **p < 0.01. Two panels including a bar graph representing the presence of various background conditions and a forest plot showing the likelihood of each complication based on LT versus ST OGC use. **a** Each condition includes three bars representing the proportion of all patients (gray bars with diagonal stripes), LT OGC users (red bars with horizontal stripes), and ST OGC users (solid black bars) with the condition. The most frequent complications are infectious or parasitic disease, hyperlipidemia, hypertension, and cataracts. **b** The aOR and 95% CI are plotted for each complication shown on the *y* axis. Most aORs are > 1, except for hyperlipidemia. The 95% CIs cross 1 for hypertension, hyperlipidemia, cataracts, glaucoma, and infectious or parasitic disease

During the entire post-index period, LT OGC users had a higher incidence of cardiovascular disease, ocular disease, and certain other disorders (Fig. 1a). Comparisons between LT and ST OGC users were significant for hypertension, heart failure, deep venous thrombosis, cataracts, osteoporosis, infectious and parasitic disease, and Cushing syndrome (all *p* < 0.01; Fig. 1a). Incidence of malignancies was also higher for LT versus ST OGC users (2.6% vs 0.8%; *p* < 0.01).

After adjustment for covariates, patients in the LT OGC group had a significantly higher incidence of heart failure, deep venous thrombosis, osteoporosis, Cushing syndrome, and malignancy than those in the ST OGC group (Fig. 1b).

### Subsequent treatment

**Fig. 2:**
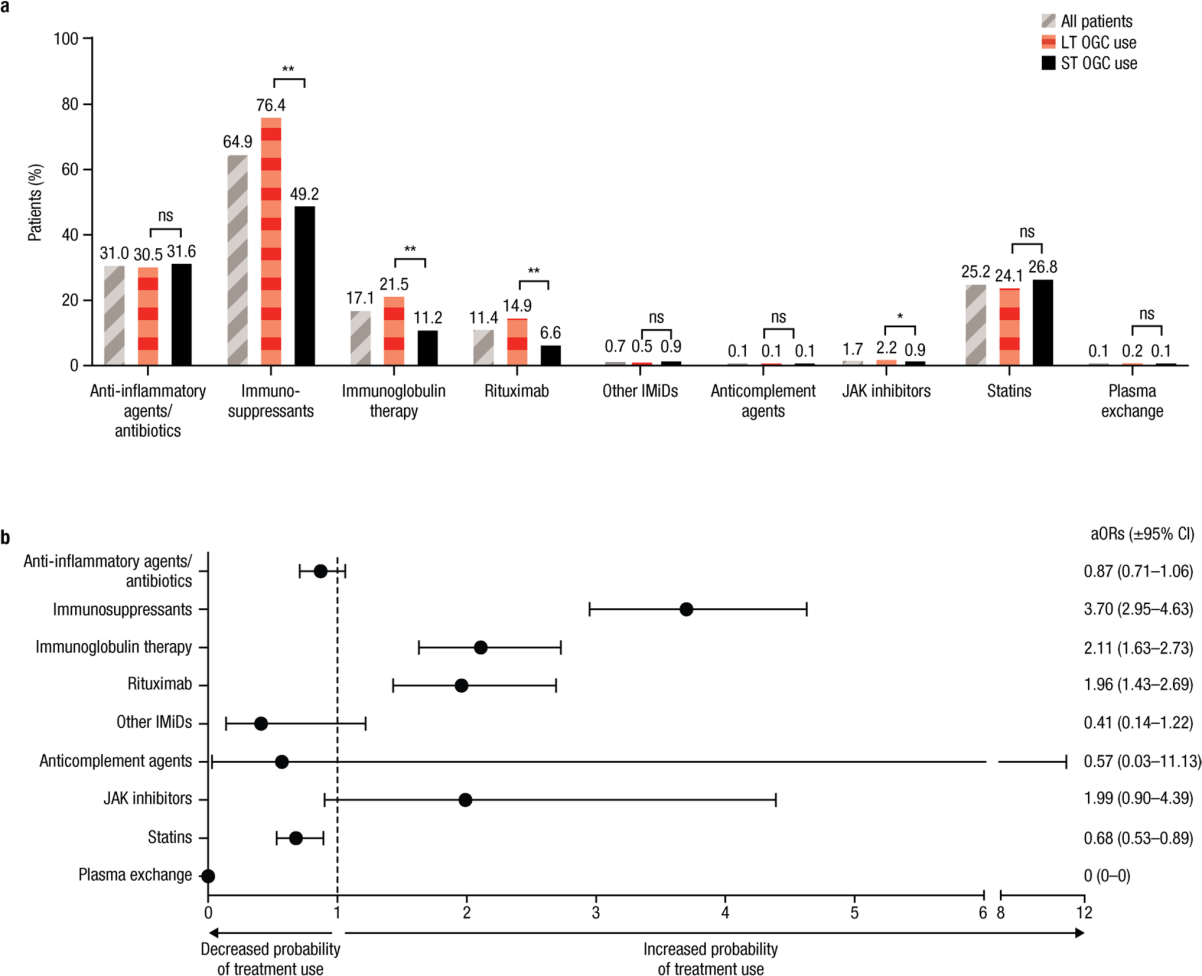
**a **Incidence of subsequent treatment use. **b **aOR (± 95% CI) in patients with LT versus ST OGC use during the 12-month post-index period. aOR, adjusted odd ratio; IMiD, immunomodulatory drug; JAK, Janus kinase; LT, long-term; NS, not significant; OGC, oral glucocorticoid; ST, short-term. *p < 0.05; **p < 0.01. Two panels including a bar graph representing the types of treatment used and a forest plot showing the likelihood of each treatment based on LT versus ST OGC use. **a** Each condition includes three bars representing the proportion of all patients (gray bars with diagonal stripes), LT OGC users (red bars with horizontal stripes), and ST OGC users (solid black bars) by treatment. The most common treatments were immunosuppressants and anti-inflammatory agents/antibiotics. **b** The aOR and 95% CI are plotted for each treatment shown on the *y* axis. The 95% CIs cross 1 for all treatments except immunosuppressants, immunoglobulin therapy, rituximab, and statins

Use of subsequent treatments during the post-index period is shown in Fig. 2a. Compared with patients in the ST OGC group, those in the LT OGC group more frequently used immunosuppressants (76.4% vs 49.2%; *p* < 0.01), immunoglobulin therapy (21.5% vs 11.2%; *p* < 0.01), rituximab (14.9% vs 6.6%; *p* < 0.01), or JAK inhibitors (2.2% vs 0.9%; *p* < 0.05), whereas similar proportions of ST and LT OGC users were treated with anti-inflammatory agents/antibiotics, other immunomodulatory drugs, anticomplement agents, statins, or plasma exchange.

After adjustment for covariates, the LT OGC group was more likely to use immunosuppressants, immunoglobulin therapy, or rituximab compared with the ST OGC group during the post-index period (Fig. 2b). In addition, the LT OGC group was less likely to use statins.

### HCRU and costs

During the 12-month post-index period, the LT OGC group had a significantly higher percentage of patients with all-cause hospitalizations (19.4% vs 11.7%; *p* < 0.01) and disease-related hospitalizations (7.6% vs 2.3%; *p* < 0.01) than the ST OGC group (Table 2). LT OGC users were also significantly more likely than ST OGC users to have all-cause prescription drug use, as well as disease-related physician visits, other outpatient services, and prescription drug use (all *p* < 0.01; Table 2). Additionally, after adjusting for baseline covariates, patients in the LT OGC group had increased odds for all-cause inpatient admission (adjusted odds ratio [aOR], 1.7; 95% confidence interval [CI], 1.3–2.2), disease-related inpatient admission (aOR, 3.8; 95% CI, 2.3–6.2), disease-related physician visits (aOR, 5.0; 95% CI, 3.5–7.2), other disease-related outpatient services (aOR, 2.5; 95% CI, 2.0–3.1), and disease-related prescription drug use (aOR, 2.5; 95% CI, 2.0–3.1) compared with the ST OGC group.

**Table 2:** HCRU of study patients during 12-month post-index period

Parameter, *n* (%)	All patients (*N* = 2280)	LT OGC use (*n* = 1313)	ST OGC use (*n* = 967)	*p* value^a^
All-cause HCRU				
Inpatient admission	368 (16.1)	255 (19.4)	113 (11.7)	< 0.01
Emergency room visit	574 (25.2)	339 (25.8)	235 (24.3)	0.41
Physician visit	2269 (99.5)	1305 (99.4)	964 (99.7)	0.31
Other outpatient services	2270 (99.6)	1309 (99.7)	961 (99.4)	0.26
Prescription drug use	2274 (99.7)	1313 (100)	961 (99.4)	< 0.01
Disease-related HCRU				
Inpatient admission	122 (5.4)	100 (7.6)	22 (2.3)	< 0.01
Emergency room visit	60 (2.6)	40 (3.0)	20 (2.1)	0.15
Physician visit	2071 (90.8)	1269 (96.6)	802 (82.9)	< 0.01
Other outpatient services	1855 (81.4)	1153 (87.8)	702 (72.6)	< 0.01
Prescription drug use	1663 (72.9)	1063 (81.0)	600 (62.0)	< 0.01

*HCRU* healthcare resource utilization, *LT* long-term, *OGC* oral glucocorticoid, *ST* short-term

^a^Comparisons refer to LT versus ST OGC exposure

**Fig. 3:**
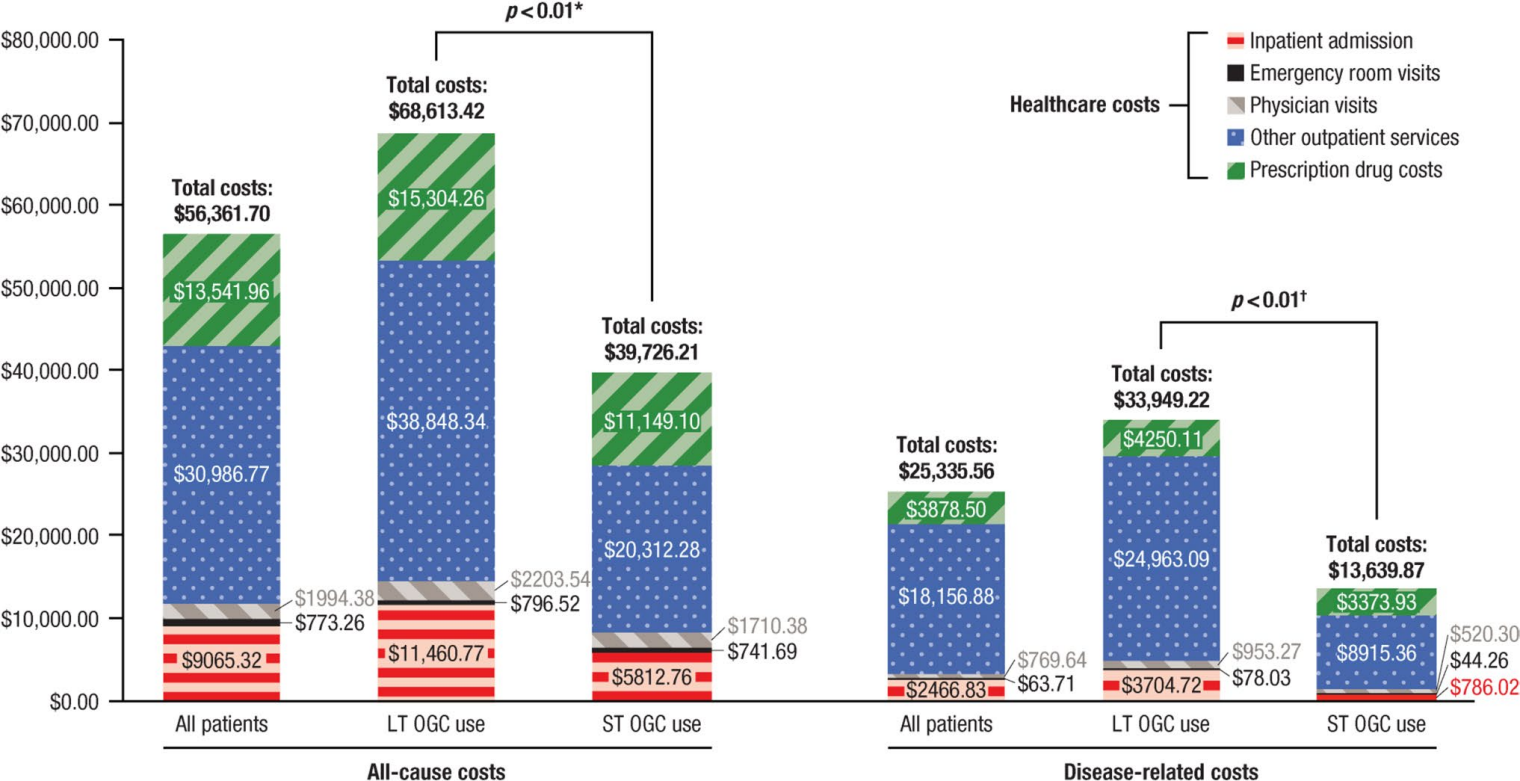
Healthcare costs during the 12-month post-index period. LT, long-term; OGC, oral glucocorticoid; ST, short-term. *p < 0.01 for total all-cause costs and all individual component cost comparisons for LT versus ST OGC use other than emergency room visits (*p* = 0.57). ^†^*p* < 0.01 for total disease-related costs and all individual component cost comparisons for LT versus ST OGC use other than emergency room visits (*p* = 0.14) and prescription drug claims (*p* = 0.34). Two sets of stacked bar graphs showing all-cause costs and disease-related costs in all patients, LT OGC users, and ST OGC users. Each bar shows the contribution of individual healthcare costs (inpatient admission [red with horizontal stripes], emergency room visits [solid black], physician visits [gray with diagonal stripes], other outpatient services [blue with dots], and prescription drug costs [green with diagonal stripes])

A higher HCRU rate among LT OGC users yielded higher costs. As shown in Fig. 3, patients in the LT OGC group incurred an average of US $68,613 in total all-cause costs during the 12-month post-index period, whereas the average all-cause costs for ST OGC users over that same period were US $39,726. In terms of disease-related costs, LT OGC users incurred a mean of US $33,949 versus US $13,640 for ST OGC users. On average, LT OGC users incurred US $30,555 (*p* < 0.01) higher adjusted total all-cause costs and US $21,311 (*p* < 0.01) higher adjusted disease-related costs than ST OGC users.

## Discussion

This retrospective cohort study is one of the few to use the most recently available administrative claims database, representing a broad commercially insured population in the USA, to evaluate the association between LT OGC use and associated medical conditions, subsequent treatment use, HCRU, and costs among patients with DM or PM.

Results from this study show that patients with DM and PM and LT OGC use had a higher rate of OGC-related medical conditions, particularly heart failure, deep venous thrombosis, osteoporosis, Cushing syndrome, and malignancy, as well as a higher rate of subsequent treatment use (eg, immunosuppressants, immunoglobulin therapy, rituximab, or statins), compared with ST OGC users. Higher all-cause and disease-related HCRU and associated costs were also incurred for LT versus ST OGC users.

The present study showed that prolonged use of OGC is linked with the occurrence of associated medical conditions of interest among patients with DM and PM, as has previously been reported for other therapeutic areas [9–14]. One systematic review of OGC exposure independent of disease area reported high rates of hypertension and fractures/osteoporosis with LT OGC use, as well as a fourfold higher risk of type 2 diabetes and hyperglycemia compared with nonusers [13]. Another focused review of studies in rheumatoid arthritis revealed various adverse events among LT OGC users, such as bone loss, cardiovascular events, and infections [21]. A systematic review of atopic dermatitis found several systemic adverse events associated with glucocorticoid use, such as osteoporosis, Cushing syndrome, and hypertension, among others [22]. Overall, most glucocorticoid treatment guidelines in autoimmune diseases recommend very low doses of OGC if LT treatment is needed, and the use of alternative steroid-sparing treatments is highly advised [13].

While prolonged use of OGC was linked with the occurrence of malignancy in this study, it should be noted that patients with IIM are known to have an increased risk of cancer, particularly within 3 years of diagnosis [23, 24]. According to a meta-analysis of studies in patients with DM or PM, the overall relative risk for developing malignancy increased approximately fivefold for patients with DM and twofold for patients with PM compared with the general population [25]. The mechanism behind cancer-associated myositis is mainly speculated to be related to malignancy-induced autoimmune disease processes; however, it remains unclear and warrants further investigation [23, 24].

In this study, the most frequently used subsequent treatments were anti-inflammatory agents/antibiotics, immunosuppressants, and immunoglobulin therapy, which is in line with previous reports from retrospective analyses in IIM. One study found that about 40% of patients with DM used immunosuppressants (specifically azathioprine, methotrexate, or mycophenolate mofetil) as their second-line treatment after OGC, and about one-fourth of patients used IVIG [26]. Another study of patients with DM found that, besides OGC, most patients used immunosuppressants (azathioprine, mycophenolate mofetil, or cyclophosphamide), and about 20% of patients used abatacept or rituximab [27]. Interestingly, one study that investigated PM and DM separately found that only 27% and 18% of patients, respectively, continued to a second-line agent (methotrexate, cyclophosphamide, azathioprine, IVIG, hydroxychloroquine, or mycophenolate mofetil) after OGC use [28]. These second-line agents, however, are associated with adverse events [29–31]. For example, the use of low-dose methotrexate was associated with increased risk for skin cancer and gastrointestinal, infectious, pulmonary, and hematologic adverse events [29]; treatment with azathioprine was associated with an increased rate of serious adverse events, including pancreatitis and leukopenia [30]; and treatment with rituximab was associated with a higher incidence of neutropenia [31]. Overall, these findings highlight the limitations of existing treatments for DM and PM and underscore the need for novel advanced treatment options with higher efficacy and fewer associated medical conditions.

The economic burden and HCRU of LT OGC use have also been previously reported. One study that explored a US population of patients with systemic lupus erythematosus found higher HCRU and costs for OGC users, particularly with LT use, compared with nonusers [18]; similar findings were observed in a study of patients with systemic lupus erythematosus in the UK [32].

There are a few studies reporting on the elevated HCRU and costs associated with IIM compared with healthy controls [6, 15, 17]; however, they are not particularly focused on the HCRU associated with LT OGC use. The results of these studies further corroborate the findings of the current study and highlight the sparsity of information in this field. Interestingly, it has been reported that most patients with IIM require second-line treatments beyond OGC to control their symptoms, further exacerbating the disease burden and increasing HCRU [12]. Taken together, this highlights the need for additional therapies for patients with IIM, including those with DM or PM, to potentially reduce the risk of OGC-associated medical conditions and the need for subsequent treatments, as well as ease the economic and HCRU strain resulting from the disease.

Results of this study should be interpreted considering certain limitations. First, as with other retrospective claims analyses, administrative claims data are collected for facilitating payment for medical services and do not contain clinical information found in medical records. For example, the lack of data regarding disease activity/severity and relevant medical history represents an important source of potential bias. Patients with severe disease and underlying inflammatory conditions are more likely to receive LT OGC treatment and additional treatments including immunosuppressants, leading to higher HCRU and costs. Second, comorbidities, DM and PM diagnoses, and OGC-associated medical conditions were identified based on ICD-10-CM codes. A diagnosis code on a medical claim is not confirmation that the patient had the condition because the code may represent a rule-out diagnosis or may be recorded incorrectly; for instance, the group of patients with a diagnosis code for PM may also have included patients with anti-synthetase syndrome, immune-mediated necrotizing myopathy, or overlap myositis, based on historical classifications. Thus, data are also subject to coding limitations and data entry errors that may create the potential for misclassification bias. This limitation was likely mitigated by requiring eligible patients to have at least two claims with diagnosis codes of DM or PM, with one diagnosis code associated with a physician specialty of interest. Third, pharmacy claims data do not necessarily indicate the actual medication taken by patients. Fourth, LT and ST OGC use was determined during the 12-month post-index period, which could have potentially affected group classification, as different treatment patterns could be observed with different observation periods (e.g., 6-month post-index period). Fifth, reported costs are specific to the payers included in the database and reflect the paid amounts of adjudicated claims to individual hospitals and providers, and other costs (e.g., indirect costs due to ST disability) were not included because relevant data cannot be captured from administrative claims; therefore, this may be an underestimation of the overall disease burden. Sixth, costs for patients aged 65 years and older could be underestimated because costs paid by other payers for this population were not included in the study. Lastly, this study was limited to individuals with commercial health coverage or private Medicare Supplemental coverage; consequently, these results may not be generalizable to patients with other types of insurance or without health insurance coverage.

## Conclusions

In conclusion, results from this retrospective study show that patients with DM or PM who were exposed to LT OGC use had a higher rate of associated medical conditions and subsequent treatment use, as well as substantially higher HCRU and economic burden. These findings provide insight to inform clinical care while highlighting the known limitations of LT OGC treatment and further supporting the unmet need among patients with DM or PM. While OGC are frequently employed as an induction treatment, prolonged use is associated with significant safety issues, and thus, a shorter OGC treatment duration may mitigate these risks and enhance patient outcomes. Disease management with more advanced treatments with advantageous safety profiles utilized in the disease course may be clinically impactful. Future research is warranted with a focus on LT treatment patterns and outcomes to improve understanding of the treatment and disease trajectories of DM and PM.
